# Iron-restricted *Mycobacterium tuberculosis* exports pathogenicity factors packed in extracellular vesicles

**DOI:** 10.1371/journal.pone.0324919

**Published:** 2025-05-30

**Authors:** Nishant Sharma, Nevadita Sharma, Ashis Biswas, Shamba Gupta, Assirbad Behura, Gloria Marcela Rodriguez

**Affiliations:** The Public Health Research Institute, New Jersey Medical School, Rutgers University, Newark, New Jersey, United States of America; Bennett University, INDIA

## Abstract

*Mycobacterium tuberculosis*, the pathogen responsible for human tuberculosis, responds to iron limitation by increasing the production of extracellular vesicles. This study examined the protein composition of induced *M. tuberculosis* extracellular membrane vesicles using chromatography coupled with mass spectrometry. The results revealed that vesicles contain key pathogenicity factors, including proteins that enhance bacterial survival, immune evasion, and inflammation. These findings deepen our understanding of the potential role of extracellular vesicles in *M. tuberculosis-*host interactions. The data can also aid in identifying new biomarkers of infection and developing vesicle-based, culture-independent TB diagnostic platforms.

## Introduction

*Mycobacterium tuberculosis (Mtb)* is an ancient pathogen that continues to cause significant morbidity and mortality worldwide. *Mtb* is responsible for over one million deaths due to tuberculosis (TB) each year. However, our understanding of its virulence strategies remains incomplete. Although immunocompromised individuals are at greater risk of developing TB disease, most people with subclinical TB are immunocompetent, highlighting *Mtb*’s ability to persist despite an active immune response [1]. Understanding the interactions between the pathogen and the host that undermine effective immunity is essential for developing urgently needed vaccines, innovative therapies targeted at either the pathogen or the host, and diagnostic tools to help control the ongoing TB pandemic.

A central feature of *Mtb*’s success as a pathogen is its remarkable ability to modify its environment, creating an intracellular niche and evading and subverting the macrophage’s antimicrobial defenses and immune functions; for this, *Mtb* is known to employ cell envelope glycolipids and secreted proteins. Furthermore, *Mtb* secretes extracellular vesicles, enabling distal communication with uninfected host cells [2–4].

Extracellular vesicles (EVs) are membrane-bound nanoparticles that encapsulate and transport a variety of biomolecules, including proteins, lipids, nucleic acids, and metabolites. Bacteria ubiquitously produce EVs, and this process is genetically regulated [5–8]. The composition of bacterial EVs is specific to different species and influenced by environmental factors [9,10]. Growing evidence indicates that EVs produced by symbiotic bacteria can promote beneficial health effects for the host [11,12], contrasting with the selective export of toxins and other virulence factors via EVs observed in many pathogenic bacteria [13–16]. EV production likely aids pathogens by enabling the simultaneous export, transport, and delivery of multiple effectors into host cells in a concentrated and protected manner [17].

Mycobacterial EVs originate from the cytoplasmic membrane and range in size from 60 to 300 nm in diameter [18]. We previously demonstrated that *Mtb* produces abundant vesicles when faced with iron restriction, a well-known stress condition prevalent in the host environment and intensified by nutritional immunity. *Mtb* generates very few vesicles under conditions of iron sufficiency [19]. Iron restriction also triggers the expression of many virulence-associated genes [20]. The hydrophobic siderophore and virulence factor, mycobactin [21], produced in response to iron limitation [22,23], is packed and exported in low-iron-induced EVs (hereafter referred to as MEVs) [19], which can support the growth of a siderophore biosynthesis mutant in low-iron medium, indicating a role for MEVs in iron acquisition [19].

Recently, we demonstrated that the upregulation of mycobacterial vesicle production is linked to enhanced expression of the iron-regulated dynamin-like proteins IniA and IniC, which are required for vesicle biogenesis [24]. Utilizing an *Mtb iniA* deletion mutant, we showed that the intracellular packing and subsequent export of bacterial antigens from infected macrophages within EVs enable intracellular *Mtb* to communicate with bystander host cells [24–26].

To improve understanding of MEV functionality during infection, we characterize the protein composition of MEVs using a quantitative proteomic approach. The results offer valuable insights into the potential role of MEV cargo in TB pathogenesis and establish a foundation for new research on vesicle-associated TB biomarkers.

## Materials and methods

### Culture conditions

*Mtb* H37Rv (ATCC) was recovered from a frozen stock on Middlebrook 7H10 agar supplemented with 0.2% glycerol, 0.5% bovine serum albumin fraction V, 0.2% dextrose, 0.085% NaCl, and 0.05% Tween-80. To induce MEV production, *Mtb* grown to logarithmic phase on 7H10 was inoculated into 1 L of minimal medium (MM) [0.1% asparagine, 0.05% KH_2_PO_4_, 0.25% Na_2_HPO_4_, 0.2% glycerol, 0.2% dextrose, and 0.085% NaCl (pH 6.8)] at an optical density (O.D_600_) of 0.1 and cultured at 37°C until reaching an O.D_600_ of 0.8. Before use, MM was treated with 5% Chelex-100 (BioRad) for 24 hours to remove traces of iron. Chelex was subsequently removed by filtration through a 0.22 μm filter (Millipore), and the medium was supplemented with 0.5 mg of ZnCl_2_ per liter, 0.1 mg of MnSO_4_ per liter, and 40 mg of MgSO_4_ per liter. This medium contains approximately 1 µM residual iron as determined by atomic absorption spectrometry. Bacterial growth was monitored based on the increase in O.D._600_ measured in an aliquot removed from the primary culture and supplemented with 0.05% Tween-80 to prevent bacterial clumping.

### MEV isolation

MEVs were isolated and purified as previously described [27]. Briefly, *Mtb* cultures were centrifuged at 3,900 × g for 15 minutes at 4°C. The culture supernatant was collected for vesicle isolation, while the bacterial cell pellet was processed to generate whole-cell protein extracts. The culture supernatant was sterilized by filtration through a 0.22 µm filter and concentrated to 25 ml using an Amicon Ultrafiltration system (Millipore) equipped with a 100-KDa exclusion membrane. The concentrate was centrifuged at 15,000 ×* *g for 15 minutes at 4°C to remove protein aggregates. MEVs were separated by ultracentrifugation at 100,000 × g for 2 hours at 4°C. MEVs were adjusted to 45% w/v iodixanol (OptiPrep, Sigma-Aldrich) by adding OptiPrep in PBS buffer to a total of 1 ml, then loaded into the bottom of a 12.5 mL ultracentrifuge tube (Beckman) and layered with decreasing percentages of OptiPrep/PBS (1 ml of a 40% concentration, 1 ml of 35%, 1 ml of 30%, 1 ml of 25%, 1 ml of 20%, and 1 ml of PBS). Samples were centrifuged (100,000 × g, 18 h, 4°C), and fractions were removed sequentially from the top of the gradient. The iodixanol was removed by dilution in PBS (25 ml) followed by centrifugation (100,000 × g, 1 hour, 4°C). Previously, we established that membrane vesicles separate in the Optiprep 30−25% fraction, as observed through visualization by electron microscopy, immunological detection of antigenic components, nanoparticle analysis, and estimation of vesicle-associated proteins and lipids [19,27]. MEVs isolated from three independent cultures were analyzed by LC-MS/MS.

### Generation of whole-cell protein extracts

The cell pellet collected from MM cultures was resuspended in 20 ml of cold PBS along with a protease inhibitor cocktail (Roche). The cells were lysed using two 1-minute pulses of bead beating (FastPrep-24 instrument, MP Biomedicals, Santa Ana, CA) with ice cooling between pulses. The lysates were centrifuged at 10,000 x g at 4°C to eliminate cell debris, and the supernatant was collected and filtered through a 0.22 µm filter.

### Proteomic analysis

#### Sample preparation.

Five micrograms of each sample were reduced and denatured by adding 10 mM DTT and heating at 60^°^C for 30 minutes. Next, the samples were alkylated with 20 mM iodoacetamide for 45 minutes at room temperature in the dark. Sequencing grade trypsin (0.2 µg, Thermo Scientific) was added, and the proteins were digested at 37^°^C overnight. Peptides were extracted twice with 5% (v/v) formic acid and 60% (v/v) acetonitrile and then dried under vacuum.

#### Liquid chromatography-tandem mass spectrometry (LC-MS/MS).

Samples were analyzed by LC-MS using Nano LC-MS/MS (Dionex Ultimate 3000 RLSCnano System, interfaced with Eclipse (ThermoFisher).

Three microliters of a 12.5 µl peptide digest per sample were loaded onto a fused silica trap column (Acclaim PepMap 100, 75 µm x 2 cm, ThermoFisher). After washing for 5 minutes at a flow rate of 5 µl/min with 0.1% TFA, the trap column was connected in line with an analytical column (Nanoease MZ peptide BEH C18, 130A, 1.7 µm, 75 µm x 250 mm, Waters) for LC-MS/MS. Peptides were fractionated at 300 nL/min using a segmented linear gradient of 4–15% B over 30 minutes (where A is 0.2% formic acid, and B is 0.16% formic acid, 80% acetonitrile), followed by 15–25% B over 40 minutes, 25–50% B over 44 minutes, and then 50–90% B over 11 minutes. Solution B returns to 4% for 5 minutes for the next run.

#### Data collection and analysis.

The eluted peptides were analyzed using the DIA (Data Independent Acquisition) workflow. The MS scan range was set from 400 to 1200, the resolution was 12,000, AGC was configured to 3E6, and the ion time was set to auto. An 8 m/z window was used to sequentially isolate (AGC 4E5 and ion time set to auto) and fragment the ions in a C-trap with a relative collision energy of 30. The MS/MS data were recorded at a resolution of 30,000.

Two samples (EV3 and CP3) were also analyzed using DDA (data-dependent acquisition) to create specialized libraries for MaxQuant analysis of DIA data. The DDA scan sequence commenced with an MS1 spectrum (Orbitrap analysis, resolution 120,000, scan range from M/Z 375–1500, automatic gain control (AGC) target 1E6, maximum injection time 100 ms). The top S (3 sec) duty cycle scheme was employed to determine the number of MS/MS events performed during each cycle. Parent ions with charges between 2 and 7 were selected for MS/MS, and a dynamic exclusion of 60 seconds was applied to prevent repeat sampling. Parent masses were isolated in the quadrupole with an isolation window of 1.2 m/z, AGC target of 1E5, and fragmented using higher-energy collisional dissociation with a normalized collision energy of 30%. The fragments were scanned in Orbitrap at a resolution of 15,000. The MS/MS scan ranges were established based on the charge state of the parent ion, with the lower limit set at 110 amu.

The DDA raw mass spectrometry data were processed with MaxQuant (version 2.4.10.0) [28] and searched against the *Mycobacterium tuberculosis* H37Rv reference proteome downloaded from UniProt using the standard label-free workflow. The search parameters were as follows: carbamidomethyl (C) (fixed), acetyl (N-term), and oxidation (M) (variable) modifications were applied. For the full-scan MS spectra (MS1), the mass error tolerance was set to 20 ppm, and for the MS/MS spectra (MS2) to 20 ppm. Trypsin was selected as a protease with a maximum of two missed cleavages. For protein identification, a minimum of one unique peptide with a peptide length of at least seven amino acids and a false discovery rate below 0.01 was required on the peptide and protein levels. The DIA data were analyzed with MaxQuant again using the MaxDIA workflow [29] with the DDA evidence.txt as a library. The match between runs function was enabled, and a time window of one minute was set. Label-free quantification was selected using iBAQ (calculated as the sum of the intensities of the identified peptides and divided by the number of observable peptides of a protein) [30].

#### Statistical analysis.

The average iBAQ value of both MEVs and cell pellets (CP) was used to calculate the fold enrichment as the ratio of iBAQ in MEVs to iBAQ in CPs; n = 3. For the fold comparison, an unpaired Student’s t-test was calculated using GraphPad Prism 9.0 software. A *t*-test value of 0.01 was considered statistically significant.

## Results

### Proteomic analysis of MEVs

To investigate MEV composition, we employed mass spectrometry and identified proteins selectively packed into vesicles by *Mtb* H37Rv under iron-limited conditions. The cell pellets from three independent low-iron cultures in the logarithmic phase were lysed to generate whole-cell protein extracts, while the culture supernatants were processed for MEV isolation. MEVs were isolated, purified, and validated as previously [19,24,27] (S1 Fig). The whole-cell protein extracts and MEV preparations underwent proteomic analysis using tryptic digestion followed by LC-MS/MS. Proteins with at least five identified peptides in MEVs (totaling 2,192) are detailed in the S1 Data Set. The relative abundance of each protein in each preparation was determined based on the quantitative MS intensities (Intensity Absolute Quantification or iBAQ), calculated as the sum of the intensities of the identified peptides divided by the number of observable peptides. The most abundant proteins (iBAQ ≥ 0.1%) (S2 Data Set) were selected for further analysis and annotation (S2 Fig). As expected for membrane EVs, this group of proteins predominantly consists of proteins previously identified in proteomic analyses of *Mtb* membrane fractions (97.5%) (S3 Fig) [31–33], and a substantial proportion of them (39.5%) had been detected in *Mtb* culture filtrates previously [32,34,35]. A total of 85 proteins showed higher abundance (>1.0-fold) in MEVs compared to cell pellets; for 32 of these, the difference reached statistical significance (S1 Table), indicating selective packaging into MEVs. A brief review revealed no apparent commonalities among the proteins enriched in MEVs. They display a wide range of molecular weights (6.8–176.9 KDa), isoelectric points (4.7–11.5), and lengths (61–456 amino acids), belonging to diverse protein families. Notably, lipoproteins comprised this group’s largest single protein family (18.7%) (S1 Table). We questioned whether the observed preference for incorporating these proteins into MEVs correlated with increased gene expression under iron limitation. To investigate this question, we analyzed published data on the dynamics of iron-regulated gene expression in *Mtb* [23,36]. Among the proteins enriched in MEVs, only PfkA is upregulated under iron limitation, indicating that selective packaging into MEVs is not solely dictated by gene expression.

According to the broad functional categorization in the mycobacterial database, Mycobrowser (https://mycobrowser.epfl.ch/genes), MEVs contain proteins from various categories: conserved hypothetical proteins (14.2%), proteins predicted to be involved in intermediate metabolism and respiration (41.4%), lipid metabolism (14.8%), cell wall and cell processes (11.7%), information pathways (11.1%), and virulence, detoxification, and adaptation (5.6%) (S4 Fig). However, a comprehensive review of recent literature revealed that MEVs pack numerous housekeeping proteins that moonlight as adaptation and pathogenicity effectors; based on these findings, we categorized and discussed the most abundant proteins in MEVs according to their potential functionality at the pathogen-host interface.

### MEV-associated proteins can promote bacterial survival

*Mtb* survives in the host by subverting lung macrophage antimicrobial defenses, including acidification, iron restriction, intoxication with heavy metals, antimicrobial peptides, reactive oxygen species (ROS), reactive nitrogen species (RNS), and lysosomal degradation [37]. Our proteomic analysis identified proteins in MEVs that can protect *Mtb* against ROS (NuoG, Rv2159c, Rv3671c, and KatG) [38–41], RNS (DlaT) [42], and toxic zinc accumulation (PacL) [43]. Additionally, MEVs contain AcpM, which inhibits phagosome maturation [44], and EsxA, a virulence effector that promotes phagosome membrane permeabilization and bacterial escape into the cytoplasm [45] (Table 1).

**Table 1:** MEV-associated proteins facilitate immune evasion.

Gene	Protein	Activity	Ref
Rv1908	KatG	Antioxidant, Catalase	[41]
Rv2159	Rv2159	Antioxidant, Peroxidase	[39]
Rv2215	DlaT	Peroxynitrite reduction	[42]
Rv2740	EphG	Epoxide hydrolysis and detoxification	[62]
Rv0798c	Cfp29	Detoxification	[63]
Rv3269	PacL1	Zn detoxification	[43]
Rv3671c	Rv3671c	Acidification an oxidative stress resistance	[40]
Rv2244	AcpM	Inhibitor of phagosome maturation	[44]
Rv3151	NuoG	Inhibits apoptosis and ROS production	[38,51]
Rv1479	MoxR1	Apoptosis suppressor	[52]
Rv0934	PstS1	Macrophage Adhesin	[64]
Rv0440	GroEL2	Adhesin, host cell death regulator	[53,60,65,66]
Rv0475	HBHA	Adhesin, host cell death regulator.	[67,68]
Rv0679	Rv0679	Adhesin	[69]
Rv3763	LpqH	Interferes with antigen presentation	[59]
Rv1411c	LprG	Interferes with antigen presentation	[60]
Rv1270	LprA	Interferes with antigen presentation	[61]
Rv3875	EsxA	Phagosome permeabilization	[45]

*Mtb*-infected macrophages initiate programmed cell death (apoptosis) as a last resort to eliminate intracellular infection [46]. In apoptosis, degraded cellular contents remain within membrane blebs. Subsequent efferocytosis of apoptotic cells eliminates *Mtb* and protects the host [47]. Virulent *Mtb* inhibits apoptosis and promotes necrotic host cell death, leading to tissue pathology and facilitating bacterial dissemination and transmission [48–50]. MEVs carry multiple proteins that regulate host cell death, including the apoptosis inhibitors NuoG [51], MoxR1 [52], and GroEL2 [53]. By inhibiting apoptosis in infected macrophages and neutrophils, these proteins delay antigen acquisition by dendritic cells and trafficking to the lymph nodes, thus hindering effective communication between the innate and adaptive immune systems. Apoptogenic proteins such as LpqH [54,55], EsxA [56], and PstS-1 [57], along with anti-autophagy factors like MoxR1, KatG, and the heparin-binding hemagglutinin protein (HBHA), were also identified in MEVs [47,58] (Table 1).

Some MEV-associated proteins help *Mtb* evade immune surveillance by directly interfering with antigen-presenting cell function and T-cell activation. For instance, the chaperone GroEL2 inhibits dendritic cell maturation, and the lipoproteins LpqH, LprG, and LprA have been demonstrated to inhibit macrophage IFN-induced MHC-II expression and antigen presentation to CD4^+^ T cells [59–61] (Table 1).

### MEV-associated proteins provoke the immune system

Evidence from various sources links mycobacterial EVs to inflammation. A mutant of *Mtb* (*virR::Tn*), which exhibits enhanced vesicle release, induced significantly more inflammation in infected mice than the parental strain [8]. Isolated vesicles derived from *Mtb* cultures injected intratracheally into mice triggered robust inflammation and exacerbated a subsequent infection with virulent *Mtb* [18]. Furthermore, EVs released by macrophages infected with an *iniA* mutant deficient in vesicle production exhibited a reduced capacity to induce proinflammatory cytokine release by uninfected macrophages compared to EVs released by wild-type *Mtb*-infected macrophages [21]. Consistent with the pro-inflammatory properties of MEVs, the data revealed that MEVs transport numerous immunostimulatory proteins, including multiple Toll-Like Receptor-2 (TLR2) agonists (LpqH, LprG, LprA, EsxA) and recognized immunodominant T cell antigens (MmsA, GroEL2, DnaK, Cfp29, RpIJ, RpsO, RpsG, and RpsC) [70–72] (Table 2).

**Table 2:** MEV-associated immunostimulatory proteins.

Gene	Protein	Activity	Reference
Rv0315	Rv0315	T cell antigen	[73]
Rv0350	DnaK	Immune activator	[74]
Rv0440	GroEL2	T cell antigen	[75]
Rv0475	HBHA	Antigenic protein	[76]
Rv0580c	Rv0580c	Antigenic protein	[77]
Rv0651	RplJ	Antigenic protein	[72]
Rv0683	RpsG	T cell antigen	[71]
Rv0707	RpsC	T cell antigen	[71]
Rv0753c	MmsA	Antigenic protein	[70]
Rv0798c	Cfp29	T cell Antigen	[78]
Rv0889c	CitA	Antigenic protein	[79]
Rv0896	GltA2	Antigenic protein	[80]
Rv0934	PstS1	Antigenic protein	[81]
Rv1270c	LprA	TLR-2 agonist	[61]
Rv1380	PyrB	Antigenic protein	[82]
Rv1411c	LprG	TLR-2 agonist	[60]
Rv1479	MoxR1	TLR4-agonist	[52]
Rv1769	Rv1769	T cell antigen	[83]
Rv1872	LldD2	Antigenic protein	[74]
Rv2032	Acg	T cell antigen	[84]
Rv2785c	RpsO	T cell antigen	[71]
Rv3418	GroES	T cell antigen	[85]
Rv3875	EsxA	T cell antigen	[86]

### MEV-associated proteins could intersect host metabolism and signaling pathways

MEVs contain various enzymes that can target host substrates. For instance, it is well known that *Mtb* utilizes host lipids to persist in infected macrophages, and it can suppress host immunity by modulating host fatty acid metabolism [87]. In this regard, intriguingly, enzymes involved in cholesterol catabolism (HsaA, Rv3520c, and Rv2263) [88] are included in MEVs alongside FabG4, a protein shown to mediate host fatty acid synthesis when expressed in eukaryotic cells [89] and FadA3, which mediates differential acetylation of host proteins in *Mtb* infected macrophages in association with increased bacterial survival in mice [90].

MEVs also contain SerC, an enzyme that enables the production of D-serine, a metabolite that interacts with WDR24 and inhibits mTORC1 activation in CD8^+^ T cells. This action indirectly affects macrophages’ ability to restrict *Mtb* replication [91]. In addition, the ATP-binding protein encoded by Rv2623 [92] and the calcium-binding proteins DesA1 [93] and SecE2 [94] can alter host cell signaling pathways. Importantly, calcium-dependent signaling in immune cells affects phagosome maturation, apoptosis, and cytokine release [95,96].

The proteases ClpP1 [97] and Rv3671c [40] may alter host cell proteostasis. At the same time, Cds1, a cysteine desulfurase, can generate H_2_S, a potent gasotransmitter linked to enhanced *Mtb* pathogenesis, suppression of host immunity, and exacerbation of TB in mice [98,99]. Further, the ribonuclease VapC10 may target host cell RNA and alter gene expression (Table 3) [100].

**Table 3:** MEV proteins that can intersect host metabolism and signaling.

Gene	Protein	Properties	Ref
Rv3520c	Rv3520c	Cholesterol utilization	[88]
Rv3570c	HsaA	Cholesterol utilization	[88]
Rv2263	Rv2263	Cholesterol utilization	[88]
Rv0242c	FAbG4	Fatty acid biosynthesis	[89]
Rv1074c	FadA3	Protein acetylation	[90]
Rv0824c	DesA1	Calcium binding protein	[93]
Rv2623	TB31.7	ATP binding protein	[92]
Rv3684	Cds1	H_2_S producing enzyme	[98]
Rv1302	MetK	SAM synthase	[62]
Rv2461c	ClpP1	Protease	[97]
Rv3671c	Rv3671c	Protease	[40]
Rv1397c	VapC10	Ribonclease	[76]

### MEVs transport host protein-interacting partners

Some proteins identified in MEVs have been shown to interact directly with host cell proteins. These include the adhesins GroEL2 and HBHA, which bind to CD43 on macrophages [65,66], and Syndecan 4 in epithelial cells, respectively. The interaction between HBHA and epithelial cells is crucial for the extrapulmonary dissemination of *Mtb* [67,68]. LpqE, SahH, and Gap bind to ubiquitin 1, IL-8, and transferrin, respectively [101–103]. LpqE, LpqH, LpqN, Rv2314c, and Rv0954 were identified in a study of the host-pathogen interactome that focused on *Mtb*-secreted proteins [104]. The data from this study indicated that these proteins could interact with multiple host proteins. The interaction between LpqN and C3 ubiquitin ligase was validated and found essential for *Mtb* virulence in macrophages [104].

### The potential of MEVs as a source of novel TB biomarkers

As nutritional immunity restricts iron from invading pathogens, we anticipated that the cargo of MEVs would reflect that of vesicles released during infection. We reviewed the data reported by Kruh-Garcia et al. to investigate this hypothesis [105]. These authors employed targeted MRM-MS to detect mycobacterial proteins in EVs isolated from the serum of individuals with active TB. They successfully identified 17 mycobacterial proteins with high confidence. MEVs contain all of these proteins (S3 Data Set), reinforcing the close relationship between the protein cargo of MEVs and that of EVs produced during infection.

## Discussion

Iron is an essential metal for *Mtb* survival. However, its bioavailability within the host is usually tightly regulated and further limited by nutritional immunity [106]. Our previous studies established that *Mtb* upregulates EV biogenesis in response to iron restriction and that very few vesicles are released during logarithmic growth in a high-iron medium [19]. In this study, we examined induced EVs (MEVs) protein components. Overall, the proteome of MEVs can be functionally linked to the distinct types of *Mtb* interactions with the immune system: evasion of the innate immune response, immune subversion, and stimulation of a robust inflammatory response and tissue pathology needed for transmission [2]. Thus, based on the protein cargo of MEVs, we postulate that EV production may aid *Mtb* in evading immune-mediated killing while exploiting the host inflammatory response to continue its life cycle. Importantly, MEVs contained *Mtb* proteins associated with EVs released in humans infected with *Mtb.* This supports the significance of MEVs as a model for EVs produced *in vivo* and, therefore, a potential source of EV-associated protein signatures of TB infection.

Consistent with their cytoplasmic membrane origin, MEVs contain multiple membrane-associated proteins and various proteins found in *Mtb* culture filtrates. Although our study is limited to three independent cultures, some proteins appeared significantly enriched in MEVs compared to whole cells, suggesting a mechanism for vesicle cargo selection. The packing of selected proteins into bacterial EVs has been demonstrated, and molecular markers for cargo inclusion have been proposed in outer membrane vesicles (OMVs) produced by Gram-negative bacteria [9]. However, in Gram-positive bacteria, the mechanisms of vesicle cargo selection remain largely unexplored. We found no correlation between the low iron-induced gene expression and the preferential inclusion of proteins into MEVs. There were no common physical properties among the enriched proteins except that many are lipoproteins. Additional studies are needed to understand the basis for selective protein packing into EVs in mycobacteria.

Our proteomic analysis indicates that *Mtb* utilizes MEVs to export proteins that promote its survival and virulence by preserving a favorable host environment and evading host cell death-related bactericidal effects [56,107]. Uncovering export via MEVs enhances our understanding of how particular pathogenicity effectors may function. For instance, until now, NuoG and GroEL, two recognized anti-apoptotic factors, were thought to affect macrophage biology indirectly, as they were believed to be restricted to the bacterial surface. The export within MEVs suggests they could directly target key phagosome-lysosome fusion and apoptosis regulators. Moreover, our findings indicate that bacterial proteins, previously thought to operate endogenously, might interact with the host upon export in MEVs. A notable example is the ATP-binding protein encoded by Rv2623. This protein regulates *Mtb* growth in mice. A mutant of Rv2623 fails to establish a chronic infection and is hypervirulent in mice and guinea pigs [92]. However, how this protein controls *Mtb* growth *in vivo* remains unclear. Our data suggest that the export within MEVs may enable Rv2623 to interface with host pathways that restrict bacterial growth.

Although seemingly paradoxical, our data indicate that MEVs carry proteins that can stimulate the immune system to fight the infection alongside proteins involved in immune evasion and bacterial survival. These include several validated TLR2 agonists and other potent immunostimulators. Activation of TLRs typically promotes immunity. However, prolonged TLR2 activation by agonists in MEVs, such as LpqH, LprG, and LprA, has been shown to induce immunosuppressive cytokines (*e.g*., IL-10) and inhibit MHC-II antigen presentation [108]. Therefore, prolonged TLR2 activation by MEVs may suppress immune responses, thereby facilitating immune evasion.

Additionally, MEVs may serve as decoys, triggering immune responses that do not aid in pathogen control. Research has shown that exporting antigens from infected macrophages within EVs diverts bacterial proteins from the canonical antigen presentation pathway and limits T cell control of *Mtb* infection [109,110]. Moreover, exporting immunologically active proteins in MEVs may benefit the pathogen by fostering an environment conducive to transmission. *Mtb* transmission necessitates cavity development, which requires a tissue-damaging immune response directed against viable bacilli or *Mtb* antigens. Hence, it is conceivable that *Mtb* utilizes MEVs to alter the balance between undermining and eliciting host inflammation to facilitate transmission.

The data highlights the diversity and complexity of the MEV proteome, including proteins that can aid the pathogen in surviving in the host and proteins that might place the pathogen at risk of elimination by the immune system. The advantages and disadvantages of bacterial vesicle production are evident from the fast-growing literature describing EVs’ activities and composition, recently reviewed by McMillan and Kuehn [17]. For instance, vesicle production enables membrane remodeling in response to environmental conditions; vesicles are often involved in biofilm formation and maintenance, facilitate nutrient acquisition, promote communication and quorum sensing, and can help disseminate genetic material and protect virulence factors. However, vesicle release requires considerable energy, can activate innate and adaptive immune responses, interfere with colonization by competing with bacteria for attachment sites, and in polymicrobial environments, benefit non-producing cheaters. Nonetheless, the evolutionary conservation of EV production among bacterial pathogens would suggest that the selective export of bacterial products in EVs should ultimately benefit the pathogen.

Our results provide a foundation for further characterization of MEV functionality and bear significant practical implications. For instance, the data identified several well-known adhesins in MEVs that may facilitate interactions between MEVs and host cells. If this is the case, it might be possible to inhibit MEVs by blocking MEV-associated adhesins. Additionally, when used as immunogens, mycobacterial EVs conferred similar protection against *Mtb* infection as BCG [111], and specific antibodies targeting MEV components identified here, such as LpqH and PstS1, have been shown to protect mice against *Mtb* infection [112]. Consequently, the immunological characterization of MEV components may assist in engineering EVs enriched with antigens that elicit a protective immune response, thereby contributing to effective vaccine development. Lastly, the overlap between the composition of MEVs and EVs present in the serum of TB patients suggests that incorporating multiple MEV-associated proteins into vesicle-based biomarker platforms could advance research in sputum culture-independent TB diagnosis. One abundant protein in MEVs, LprG, has already demonstrated great promise as a biomarker of TB infection in the pediatric population, a group of patients historically challenging to diagnose with sputum-based assays [113].

In conclusion, although our study is limited to vesicles produced by iron-limited bacteria in culture, it highlighted the diversity and complexity of the MEV proteome, the selective nature of protein incorporation into vesicles, and the potential of MEVs in a plethora of Mtb-host cell interactions. The data indicate that the production of MEVs could benefit the pathogen by contributing to stress relief, nutrient acquisition, virulence, and immune evasion. However, MEVs can also trigger host protective immune activation. This suggests that balancing the beneficial and detrimental activities of MEVs may influence the outcome of TB infection. Lastly, the observed overlap between proteins in MEVs and those detected in EVs in the serum of TB patients suggests that the proteome of MEVs can be a good resource for investigating novel vesicle-associated TB biomarkers and developing culture-independent diagnostic tools.

## Supporting information

S1 Data Data Set: Proteins with five or more identified peptides in the three replicates. The number of peptides is indicated.
(XLSX)

S2 Data Data Set: Most abundant proteins selected based on an iBAQ value of 0.1% or higher. (XLSX)

S3 Data Data Set. Proteins in MEVs that were found in EVs released during TB infection. (XLSX)

S1 Fig Characterization of MEVs. Purified MEVs were analyzed by electron microscopy, and size distribution was validated by nanoparticle tracking analysis (NTA) conducted using ZetaView (Particle Metrix).
(TIF)

S2 Fig Proteomic data analysis and annotation Process. (PDF)

S3 Fig Cellular localization of the most abundant proteins in MEVs. (TIFF)

S4 Fig Functional categorization of MEV proteins according to Mycobrowser. (TIFF)

S1 Table Proteins enriched in MEVs compared to whole cells. (DOCX)

## References

[1] Ernst JD. The immunological life cycle of tuberculosis. Nat Rev Immunol. 2012;12(8):581–91. doi: 10.1038/nri3259 22790178

[2] Chandra P, Grigsby S, Philips J. Immune evasion and provocation by mycobacterium tuberculosis. Nat Rev Microbiol. 2022;20(12):750–66. 35879556 10.1038/s41579-022-00763-4 PMC9310001

[3] Gupta S, Rodriguez GM. Mycobacterial extracellular vesicles and host pathogen interactions. Pathog Dis. 2018;76(4):fty031. doi: 10.1093/femspd/fty031 29722822 PMC5930244

[4] Palacios A, Gupta S, Rodriguez GM, Prados-Rosales R. Extracellular vesicles in the context of Mycobacterium tuberculosis infection. Mol Immunol. 2021;133:175–81. doi: 10.1016/j.molimm.2021.02.010 33743266 PMC9909588

[5] Kitagawa R, Takaya A, Ohya M, Mizunoe Y, Takade A, Yoshida S, et al. Biogenesis of Salmonella enterica serovar typhimurium membrane vesicles provoked by induction of PagC. J Bacteriol. 2010;192(21):5645–56. doi: 10.1128/JB.00590-10 20802043 PMC2953678

[6] Kulp AJ, Sun B, Ai T, Manning AJ, Orench-Rivera N, Schmid AK, et al. Genome-Wide Assessment of Outer Membrane Vesicle Production in Escherichia coli. PLoS One. 2015;10(9):e0139200. doi: 10.1371/journal.pone.0139200 26406465 PMC4583269

[7] Nevermann J, Silva A, Otero C, Oyarzun D, Barrera B, Gil F. Identification of genes involved in biogenesis of outer membrane vesicles (OMVs) in Salmonella enterica serovar Typhi. Front Microbiol. 2019;10:104. 30778340 10.3389/fmicb.2019.00104 PMC6369716

[8] Rath P, Huang C, Wang T, Wang T, Li H, Prados-Rosales R, et al. Genetic regulation of vesiculogenesis and immunomodulation in Mycobacterium tuberculosis. Proc Natl Acad Sci U S A. 2013;110(49):E4790-7. doi: 10.1073/pnas.1320118110 24248369 PMC3856836

[9] Orench-Rivera N, Kuehn MJ. Differential Packaging Into Outer Membrane Vesicles Upon Oxidative Stress Reveals a General Mechanism for Cargo Selectivity. Front Microbiol. 2021;12:561863. doi: 10.3389/fmicb.2021.561863 34276573 PMC8284480

[10] Orench-Rivera N, Kuehn MJ. Environmentally controlled bacterial vesicle-mediated export. Cell Microbiol. 2016;18(11):1525–36. doi: 10.1111/cmi.12676 27673272 PMC5308445

[11] Beliakoff RE, Gonzalez CF, Lorca GL. Bile promotes Lactobacillus johnsonii N6.2 extracellular vesicle production with conserved immunomodulatory properties. Sci Rep. 2024;14(1):12272. doi: 10.1038/s41598-024-62843-0 38806562 PMC11133329

[12] Cañas M-A, Giménez R, Fábrega M-J, Toloza L, Baldomà L, Badia J. Outer Membrane Vesicles from the Probiotic Escherichia coli Nissle 1917 and the Commensal ECOR12 Enter Intestinal Epithelial Cells via Clathrin-Dependent Endocytosis and Elicit Differential Effects on DNA Damage. PLoS One. 2016;11(8):e0160374. doi: 10.1371/journal.pone.0160374 27487076 PMC4972321

[13] Kolling GL, Matthews KR. Export of virulence genes and Shiga toxin by membrane vesicles of Escherichia coli O157:H7. Appl Environ Microbiol. 1999;65(5):1843–8. doi: 10.1128/AEM.65.5.1843-1848.1999 10223967 PMC91264

[14] Horstman AL, Kuehn MJ. Enterotoxigenic Escherichia coli secretes active heat-labile enterotoxin via outer membrane vesicles. J Biol Chem. 2000;275(17):12489–96. doi: 10.1074/jbc.275.17.12489 10777535 PMC4347834

[15] Chatterjee D, Chaudhuri K. Association of cholera toxin with Vibrio cholerae outer membrane vesicles which are internalized by human intestinal epithelial cells. FEBS Lett. 2011;585(9):1357–62. doi: 10.1016/j.febslet.2011.04.017 21510946

[16] Rompikuntal P, Thay B, Khan M, Alanko J, Penttinen A, Asikainen S. Perinuclear localization of internalized outer membrane vesicles carrying active cytolethal distending toxin from Aggregatibacter actinomycetemcomitans. Infect Immun. 2012;80(1):31–42. 22025516 10.1128/IAI.06069-11 PMC3255663

[17] McMillan HM, Kuehn MJ. The extracellular vesicle generation paradox: a bacterial point of view. EMBO J. 2021;40(21):e108174. doi: 10.15252/embj.2021108174 34636061 PMC8561641

[18] Prados-Rosales R, Baena A, Martinez LR, Luque-Garcia J, Kalscheuer R, Veeraraghavan U, et al. Mycobacteria release active membrane vesicles that modulate immune responses in a TLR2-dependent manner in mice. J Clin Invest. 2011;121(4):1471–83. doi: 10.1172/JCI44261 21364279 PMC3069770

[19] Prados-Rosales R, Weinrick BC, Piqué DG, Jacobs WR Jr, Casadevall A, Rodriguez GM. Role for *Mycobacterium tuberculosis* membrane vesicles in iron acquisition. J Bacteriol. 2014;196(6):1250–6. doi: 10.1128/JB.01090-13 24415729 PMC3957709

[20] Rodriguez GM, Sharma N, Biswas A, Sharma N. The Iron Response of Mycobacterium tuberculosis and Its Implications for Tuberculosis Pathogenesis and Novel Therapeutics. Front Cell Infect Microbiol. 2022;12:876667. doi: 10.3389/fcimb.2022.876667 35646739 PMC9132128

[21] Reddy P, Puri R, Chauhan P, Kar R, Rohilla A, Khera A. Disruption of mycobactin biosynthesis leads to attenuation of *Mycobacterium tuberculosis* for growth and virulence. J Infect Dis. 2013. 10.1093/infdis/jit250 23788726

[22] Sritharan M, Ratledge C. Co-ordinated expression of the components of iron transport (mycobactin, exochelin and envelope proteins) inMycobacterium neoaurum. FEMS Microbiology Letters. 1989;60(2):183–5. doi: 10.1111/j.1574-6968.1989.tb03442.x 2777064

[23] Rodriguez GM, Voskuil MI, Gold B, Schoolnik GK, Smith I. *IdeR*, an essential gene in *Mycobacterium tuberculosis*: role of IdeR in iron-dependent gene expression, iron metabolism, and oxidative stress response. Infect Immun. 2002;70(7):3371–81. 12065475 10.1128/IAI.70.7.3371-3381.2002 PMC128082

[24] Gupta S, Bhagavathula M, Sharma V, Sharma N, Sharma N, Biswas A, et al. Dynamin-like proteins mediate extracellular vesicle secretion in Mycobacterium tuberculosis. EMBO Rep. 2023;24(6):e55593. doi: 10.15252/embr.202255593 37079766 PMC10240201

[25] Athman JJ, Wang Y, McDonald DJ, Boom WH, Harding CV, Wearsch PA. Bacterial Membrane Vesicles Mediate the Release of Mycobacterium tuberculosis Lipoglycans and Lipoproteins from Infected Macrophages. J Immunol. 2015;195(3):1044–53. doi: 10.4049/jimmunol.1402894 26109643 PMC4506856

[26] Athman JJ, Sande OJ, Groft SG, Reba SM, Nagy N, Wearsch PA, et al. Mycobacterium tuberculosis Membrane Vesicles Inhibit T Cell Activation. J Immunol. 2017;198(5):2028–37. doi: 10.4049/jimmunol.1601199 28122965 PMC5322216

[27] Gupta S, Rodriguez G. Isolation and characterization of extracellular vesicles produced by iron-limited mycobacteria. J Vis Exp. 2019;2019(152). 10.3791/60359 31736487

[28] Cox J, Mann M. Maxquant enables high peptide identification rates individualized p.p.b.-range mass accuracies and proteome-wide protein quantification. Nat Biotechnol. 2008;26(12):1367–72. 19029910 10.1038/nbt.1511

[29] Sinitcyn P, Hamzeiy H, Salinas Soto F, Itzhak D, McCarthy F, Wichmann C, et al. MaxDIA enables library-based and library-free data-independent acquisition proteomics. Nat Biotechnol. 2021;39(12):1563–73. doi: 10.1038/s41587-021-00968-7 34239088 PMC8668435

[30] Schwanhäusser B, Busse D, Li N, Dittmar G, Schuchhardt J, Wolf J, et al. Global quantification of mammalian gene expression control. Nature. 2011;473(7347):337–42. doi: 10.1038/nature10098 21593866

[31] Gu S, Chen J, Dobos KM, Bradbury EM, Belisle JT, Chen X. Comprehensive proteomic profiling of the membrane constituents of a Mycobacterium tuberculosis strain. Mol Cell Proteomics. 2003;2(12):1284–96. doi: 10.1074/mcp.M300060-MCP200 14532352

[32] de Souza GA, Leversen NA, Målen H, Wiker HG. Bacterial proteins with cleaved or uncleaved signal peptides of the general secretory pathway. J Proteomics. 2011;75(2):502–10. doi: 10.1016/j.jprot.2011.08.016 21920479

[33] Xiong Y, Chalmers MJ, Gao FP, Cross TA, Marshall AG. Identification of Mycobacterium tuberculosis H37Rv integral membrane proteins by one-dimensional gel electrophoresis and liquid chromatography electrospray ionization tandem mass spectrometry. J Proteome Res. 2005;4(3):855–61. doi: 10.1021/pr0500049 15952732

[34] Mattow J, Schaible UE, Schmidt F, Hagens K, Siejak F, Brestrich G. Comparative proteome analysis of culture supernatant proteins from virulent mycobacterium tuberculosis h37rv and attenuated m. bovis bcg copenhagen. Electrophoresis. 2003;24(19–20):3405–20. 14595687 10.1002/elps.200305601

[35] Målen H, Berven FS, Fladmark KE, Wiker HG. Comprehensive analysis of exported proteins from Mycobacterium tuberculosis H37Rv. Proteomics. 2007;7(10):1702–18. doi: 10.1002/pmic.200600853 17443846

[36] Kurthkoti K, Amin H, Marakalala M, Ghanny S, Subbian S, Sakatos A. The capacity of Mycobacterium tuberculosis to survive iron starvation might enable it to persist in iron-deprived microenvironments of human granulomas. mBio. 2017;8(4). 10.1128/mBio.01092-17 PMC5559634 28811344

[37] Weiss G, Schaible UE. Macrophage defense mechanisms against intracellular bacteria. Immunol Rev. 2015;264(1):182–203. doi: 10.1111/imr.12266 25703560 PMC4368383

[38] Miller JL, Velmurugan K, Cowan MJ, Briken V. The type I NADH dehydrogenase of Mycobacterium tuberculosis counters phagosomal NOX2 activity to inhibit TNF-alpha-mediated host cell apoptosis. PLoS Pathog. 2010;6(4):e1000864. doi: 10.1371/journal.ppat.1000864 20421951 PMC2858756

[39] Bhargavi G, Singh AK, Deenadayalan A, Ponnuraja C, Patil SA, Palaniyandi K. Role of a Putative Alkylhydroperoxidase Rv2159c in the Oxidative Stress Response and Virulence of Mycobacterium tuberculosis. Pathogens. 2022;11(6):684. doi: 10.3390/pathogens11060684 35745538 PMC9227533

[40] Biswas T, Small J, Vandal O, Odaira T, Deng H, Ehrt S, et al. Structural insight into serine protease Rv3671c that Protects M. tuberculosis from oxidative and acidic stress. Structure. 2010;18(10):1353–63. doi: 10.1016/j.str.2010.06.017 20947023 PMC2955984

[41] Ng VH, Cox JS, Sousa AO, MacMicking JD, McKinney JD. Role of KatG catalase-peroxidase in mycobacterial pathogenesis: countering the phagocyte oxidative burst. Mol Microbiol. 2004;52(5):1291–302. doi: 10.1111/j.1365-2958.2004.04078.x 15165233

[42] Shi S, Ehrt S. Dihydrolipoamide acyltransferase is critical for Mycobacterium tuberculosis pathogenesis. Infect Immun. 2006;74(1):56–63. doi: 10.1128/IAI.74.1.56-63.2006 16368957 PMC1346611

[43] Boudehen Y-M, Faucher M, Maréchal X, Miras R, Rech J, Rombouts Y, et al. Mycobacterial resistance to zinc poisoning requires assembly of P-ATPase-containing membrane metal efflux platforms. Nat Commun. 2022;13(1):4731. doi: 10.1038/s41467-022-32085-7 35961955 PMC9374683

[44] Paik S, Kim K, Kim I, Kim Y, Kim H, Choi S. Mycobacterial acyl carrier protein suppresses tfeb activation and upregulates mir-155 to inhibit host defense. Front Immunol. 2022;13:946929. 36248815 10.3389/fimmu.2022.946929 PMC9559204

[45] Augenstreich J, Arbues A, Simeone R, Haanappel E, Wegener A, Sayes F, et al. ESX-1 and phthiocerol dimycocerosates of Mycobacterium tuberculosis act in concert to cause phagosomal rupture and host cell apoptosis. Cell Microbiol. 2017;19(7):10.1111/cmi.12726. doi: 10.1111/cmi.12726 28095608

[46] Behar SM, Martin CJ, Booty MG, Nishimura T, Zhao X, Gan H-X, et al. Apoptosis is an innate defense function of macrophages against Mycobacterium tuberculosis. Mucosal Immunol. 2011;4(3):279–87. doi: 10.1038/mi.2011.3 21307848 PMC3155700

[47] Martin CJ, Booty MG, Rosebrock TR, Nunes-Alves C, Desjardins DM, Keren I, et al. Efferocytosis is an innate antibacterial mechanism. Cell Host Microbe. 2012;12(3):289–300. doi: 10.1016/j.chom.2012.06.010 22980326 PMC3517204

[48] Amaral EP, Costa DL, Namasivayam S, Riteau N, Kamenyeva O, Mittereder L, et al. A major role for ferroptosis in Mycobacterium tuberculosis-induced cell death and tissue necrosis. J Exp Med. 2019;216(3):556–70. doi: 10.1084/jem.20181776 30787033 PMC6400546

[49] Sun J, Siroy A, Lokareddy RK, Speer A, Doornbos KS, Cingolani G, et al. The tuberculosis necrotizing toxin kills macrophages by hydrolyzing NAD. Nat Struct Mol Biol. 2015;22(9):672–8. doi: 10.1038/nsmb.3064 26237511 PMC4560639

[50] Upadhyay S, Mittal E, Philips JA. Tuberculosis and the art of macrophage manipulation. Pathog Dis. 2018;76(4):fty037. doi: 10.1093/femspd/fty037 29762680 PMC6251593

[51] Velmurugan K, Chen B, Miller JL, Azogue S, Gurses S, Hsu T, et al. Mycobacterium tuberculosis nuoG is a virulence gene that inhibits apoptosis of infected host cells. PLoS Pathog. 2007;3(7):e110. doi: 10.1371/journal.ppat.0030110 17658950 PMC1924871

[52] Quadir N, Shariq M, Sheikh JA, Singh J, Sharma N, Hasnain SE, et al. Mycobacterium tuberculosis protein MoxR1 enhances virulence by inhibiting host cell death pathways and disrupting cellular bioenergetics. Virulence. 2023;14(1):2180230. doi: 10.1080/21505594.2023.2180230 36799069 PMC9980616

[53] Joseph S, Yuen A, Singh V, Hmama Z. Mycobacterium tuberculosis cpn60.2 (groel2) blocks macrophage apoptosis via interaction with mitochondrial mortalin. Biol Open. 2017;6(4):481–8. 28288970 10.1242/bio.023119 PMC5399554

[54] Ciaramella A, Martino A, Cicconi R, Colizzi V, Fraziano M. Mycobacterial 19-kDa lipoprotein mediates Mycobacterium tuberculosis-induced apoptosis in monocytes/macrophages at early stages of infection. Cell Death Differ. 2000;7(12):1270–2. doi: 10.1038/sj.cdd.4400761 11270362

[55] Ciaramella A, Cavone A, Santucci MB, Garg SK, Sanarico N, Bocchino M, et al. Induction of apoptosis and release of interleukin-1 beta by cell wall-associated 19-kDa lipoprotein during the course of mycobacterial infection. J Infect Dis. 2004;190(6):1167–76. doi: 10.1086/423850 15319868

[56] Srinivasan L, Ahlbrand S, Briken V. Interaction of Mycobacterium tuberculosis with host cell death pathways. Cold Spring Harbor perspectives in medicine. 2014;4(8). 10.1101/cshperspect.a022459 PMC4109581 24968864

[57] Sanchez A, Espinosa P, Esparza MA, Colon M, Bernal G, Mancilla R. Mycobacterium tuberculosis 38-kDa lipoprotein is apoptogenic for human monocyte-derived macrophages. Scand J Immunol. 2009;69(1):20–8. doi: 10.1111/j.1365-3083.2008.02193.x 19140873

[58] Siregar TAP, Prombutara P, Kanjanasirirat P, Kunkaew N, Tubsuwan A, Boonmee A, et al. The autophagy-resistant Mycobacterium tuberculosis Beijing strain upregulates KatG to evade starvation-induced autophagic restriction. Pathog Dis. 2022;80(1):ftac004. doi: 10.1093/femspd/ftac004 35038342 PMC8829027

[59] Stewart GR, Wilkinson KA, Newton SM, Sullivan SM, Neyrolles O, Wain JR, et al. Effect of deletion or overexpression of the 19-kilodalton lipoprotein Rv3763 on the innate response to Mycobacterium tuberculosis. Infect Immun. 2005;73(10):6831–7. doi: 10.1128/IAI.73.10.6831-6837.2005 16177361 PMC1230982

[60] Gehring AJ, Dobos KM, Belisle JT, Harding CV, Boom WH. Mycobacterium tuberculosis LprG (Rv1411c): a novel TLR-2 ligand that inhibits human macrophage class II MHC antigen processing. J Immunol. 2004;173(4):2660–8. 15294983 10.4049/jimmunol.173.4.2660

[61] Pecora ND, Gehring AJ, Canaday DH, Boom WH, Harding CV. Mycobacterium tuberculosis LprA is a lipoprotein agonist of TLR2 that regulates innate immunity and APC function. J Immunol. 2006;177(1):422–9. doi: 10.4049/jimmunol.177.1.422 16785538

[62] UniProt C. UniProt: the universal protein knowledgebase in 2025. Nucleic Acids Res. 2024. 10.1093/nar/gkae1010 PMC11701636 39552041

[63] Lien KA, Dinshaw K, Nichols RJ, Cassidy-Amstutz C, Knight M, Singh R, et al. A nanocompartment system contributes to defense against oxidative stress in Mycobacterium tuberculosis. Elife. 2021;10:e74358. doi: 10.7554/eLife.74358 34751132 PMC8635971

[64] Esparza M, Palomares B, García T, Espinosa P, Zenteno E, Mancilla R. PstS-1, the 38-kDa Mycobacterium tuberculosis glycoprotein, is an adhesin, which binds the macrophage mannose receptor and promotes phagocytosis. Scand J Immunol. 2015;81(1):46–55. doi: 10.1111/sji.12249 25359607

[65] Hickey TBM, Thorson LM, Speert DP, Daffé M, Stokes RW. Mycobacterium tuberculosis Cpn60.2 and DnaK are located on the bacterial surface, where Cpn60.2 facilitates efficient bacterial association with macrophages. Infect Immun. 2009;77(8):3389–401. doi: 10.1128/IAI.00143-09 19470749 PMC2715658

[66] Hickey TB, Ziltener HJ, Speert DP, Stokes RW. Mycobacterium tuberculosis employs Cpn60.2 as an adhesin that binds CD43 on the macrophage surface. Cell Microbiol. 2010;12(11):1634–47. 20633027 10.1111/j.1462-5822.2010.01496.x

[67] Zimmermann N, Saiga H, Houthuys E, Moura-Alves P, Koehler A, Bandermann S, et al. Syndecans promote mycobacterial internalization by lung epithelial cells. Cell Microbiol. 2016;18(12):1846–56. doi: 10.1111/cmi.12627 27279134

[68] Pethe K, Alonso S, Biet F, Delogu G, Brennan MJ, Locht C, et al. The heparin-binding haemagglutinin of M. tuberculosis is required for extrapulmonary dissemination. Nature. 2001;412(6843):190–4. doi: 10.1038/35084083 11449276

[69] Cifuentes DP, Ocampo M, Curtidor H, Vanegas M, Forero M, Patarroyo ME, et al. Mycobacterium tuberculosis Rv0679c protein sequences involved in host-cell infection: potential TB vaccine candidate antigen. BMC Microbiol. 2010;10:109. doi: 10.1186/1471-2180-10-109 20388213 PMC2873487

[70] Kim J-S, Kim WS, Choi H-H, Kim HM, Kwon KW, Han SJ, et al. Mycobacterium tuberculosis MmsA, a novel immunostimulatory antigen, induces dendritic cell activation and promotes Th1 cell-type immune responses. Cell Immunol. 2015;298(1–2):115–25. doi: 10.1016/j.cellimm.2015.10.005 26507911

[71] Kennedy SC, Johnson AJ, Bharrhan S, Lindestam Arlehamn CS, Xu J, Garforth SJ, et al. Identification of Mycobacterial Ribosomal Proteins as Targets for CD4+ T Cells That Enhance Protective Immunity in Tuberculosis. Infect Immun. 2018;86(9):e00009-18. doi: 10.1128/IAI.00009-18 29891545 PMC6105890

[72] Johnson AJ, Kennedy SC, Lindestam Arlehamn CS, Goldberg MF, Saini NK, Xu J. Identification of mycobacterial rplj/l10 and rpsa/s1 proteins as novel targets for cd4( ) t cells. Infect Immun. 2017;85(4). 10.1128/IAI.01023-16 PMC5364311 28115505

[73] Byun E, Kim W, Shin A, Kim J, Whang J, Won C. Rv0315, a novel immunostimulatory antigen of Mycobacterium tuberculosis, activates dendritic cells and drives Th1 immune responses. J Mol Med. 2012;90(3):285–98. 21993523 10.1007/s00109-011-0819-2

[74] Li Y, Zeng J, Shi J, Wang M, Rao M, Xue C, et al. A proteome-scale identification of novel antigenic proteins in Mycobacterium tuberculosis toward diagnostic and vaccine development. J Proteome Res. 2010;9(9):4812–22. doi: 10.1021/pr1005108 20690665

[75] Kaufmann SH, Väth U, Thole JE, Van Embden JD, Emmrich F. Enumeration of T cells reactive with Mycobacterium tuberculosis organisms and specific for the recombinant mycobacterial 64-kDa protein. Eur J Immunol. 1987;17(3):351–7. doi: 10.1002/eji.1830170308 3106059

[76] Masungi C, Temmerman S, Van Vooren J, Drowart A, Pethe K, Menozzi F. Differential t and b cell responses against mycobacterium tuberculosis heparin-binding hemagglutinin adhesin in infected healthy individuals and patients with tuberculosis. J Infect Dis. 2002;185(4):513–20. 11865404 10.1086/338833

[77] Ji L, Fu Y, Xiong S. Chimeric antigen carried by extracellular vesicles induces stronger protective immunity against Mycobacterium tuberculosis infection. Immunobiology. 2024;229(5):152834. doi: 10.1016/j.imbio.2024.152834 38968836

[78] Nayak K, Jing L, Russell RM, Davies DH, Hermanson G, Molina DM, et al. Identification of novel Mycobacterium tuberculosis CD4 T-cell antigens via high throughput proteome screening. Tuberculosis (Edinb). 2015;95(3):275–87. doi: 10.1016/j.tube.2015.03.001 25857935 PMC4618479

[79] Karuppusamy S, Mutharia L, Kelton D, Karrow N, Kirby G. Identification of antigenic proteins from Mycobacterium avium subspecies paratuberculosis cell envelope by comparative proteomic analysis. Microbiology (Reading). 2018;164(3):322–37. doi: 10.1099/mic.0.000606 29458660

[80] Sharma N, Khandelwal V, Kumar S, Joshi B, Mohanty KK. Immunological depiction of synthetic B-cell epitopes of Mycobacterium tuberculosis. Int J Mycobacteriol. 2023;12(4):380–7. doi: 10.4103/ijmy.ijmy_187_23 38149531

[81] Espitia C, Cervera I, González R, Mancilla R. A 38-kD Mycobacterium tuberculosis antigen associated with infection. Its isolation and serologic evaluation. Clin Exp Immunol. 1989;77(3):373–7. 2478322 PMC1542060

[82] Kerns PW, Ackhart DF, Basaraba RJ, Leid JG, Shirtliff ME. Mycobacterium tuberculosis pellicles express unique proteins recognized by the host humoral response. Pathog Dis. 2014;70(3):347–58. doi: 10.1111/2049-632X.12142 24453174 PMC4363115

[83] Lu M, Xia ZY, Bao L. A Mycobacterium bovis BCG-naked DNA prime-boost vaccination strategy induced CD4⁺ and CD8⁺ T-cell response against Mycobacterium tuberculosis immunogens. J Immunol Res. 2014;2014:395626. doi: 10.1155/2014/395626 24741595 PMC3987877

[84] Singh S, Sharma M, Chaudhry A, Sharma S. Rv2626c and Rv2032 activate TH1 response and downregulate regulatory T cells in peripheral blood mononuclear cells of tuberculosis patients. Comp Immunol Microbiol Infect Dis. 2019;62:46–53. doi: 10.1016/j.cimid.2018.11.016 30711045

[85] Barnes PF, Mehra V, Rivoire B, Fong SJ, Brennan PJ, Voegtline MS, et al. Immunoreactivity of a 10-kDa antigen of Mycobacterium tuberculosis. J Immunol. 1992;148(6):1835–40. doi: 10.4049/jimmunol.148.6.1835 1371791

[86] Ravn P, Demissie A, Eguale T, Wondwosson H, Lein D, Amoudy HA, et al. Human T cell responses to the ESAT-6 antigen from Mycobacterium tuberculosis. J Infect Dis. 1999;179(3):637–45. doi: 10.1086/314640 9952370

[87] Yang H, Wang F, Guo X, Liu F, Liu Z, Wu X, et al. Interception of host fatty acid metabolism by mycobacteria under hypoxia to suppress anti-TB immunity. Cell Discov. 2021;7(1):90. doi: 10.1038/s41421-021-00301-1 34608123 PMC8490369

[88] van Wyk R, van Wyk M, Mashele S, Nelson D, Syed K. Comprehensive comparative analysis of cholesterol catabolic genes/proteins in mycobacterial species. Int J Mol Sci. 2019;20(5). 10.3390/ijms20051032 PMC6429209 30818787

[89] Gurvitz A. The essential mycobacterial genes, fabG1 and fabG4, encode 3-oxoacyl-thioester reductases that are functional in yeast mitochondrial fatty acid synthase type 2. Mol Genet Genomics. 2009;282(4):407–16. doi: 10.1007/s00438-009-0474-2 19685079 PMC2746893

[90] Duan Y, Dong J, Shi Y, Jia H, Li Z, Xing A, et al. Effects of acetyltransferase fadA3 on acetylation of host protein and in vivo survival of Mycobacterium tuberculosis. Chin J Antituberc. 2023;45(4):391–400.

[91] Cheng H, Ji Z, Wang Y, Li S, Tang T, Wang F, et al. Mycobacterium tuberculosis produces D-serine under hypoxia to limit CD8+ T cell-dependent immunity in mice. Nat Microbiol. 2024;9(7):1856–72. doi: 10.1038/s41564-024-01701-1 38806671 PMC11222154

[92] Drumm JE, Mi K, Bilder P, Sun M, Lim J, Bielefeldt-Ohmann H, et al. Mycobacterium tuberculosis universal stress protein Rv2623 regulates bacillary growth by ATP-Binding: requirement for establishing chronic persistent infection. PLoS Pathog. 2009;5(5):e1000460. doi: 10.1371/journal.ppat.1000460 19478878 PMC2682197

[93] Yeruva VC, Savanagouder M, Khandelwal R, Kulkarni A, Sharma Y, Raghunand TR. The Mycobacterium tuberculosis desaturase DesA1 (Rv0824c) is a Ca2+ binding protein. Biochem Biophys Res Commun. 2016;480(1):29–35. doi: 10.1016/j.bbrc.2016.10.014 27721064

[94] Arockiasamy A, Aggarwal A, Savva CG, Holzenburg A, Sacchettini JC. Crystal structure of calcium dodecin (Rv0379), from Mycobacterium tuberculosis with a unique calcium-binding site. Protein Sci. 2011;20(5):827–33. doi: 10.1002/pro.607 21370306 PMC3125867

[95] Malik ZA, Iyer SS, Kusner DJ. Mycobacterium tuberculosis phagosomes exhibit altered calmodulin-dependent signal transduction: contribution to inhibition of phagosome-lysosome fusion and intracellular survival in human macrophages. J Immunol. 2001;166(5):3392–401. doi: 10.4049/jimmunol.166.5.3392 11207296

[96] Malik ZA, Denning GM, Kusner DJ. Inhibition of Ca(2 ) signaling by Mycobacterium tuberculosis is associated with reduced phagosome-lysosome fusion and increased survival within human macrophages. J Exp Med. 2000;191(2):287–302. 10637273 10.1084/jem.191.2.287 PMC2195750

[97] Ollinger J, O’Malley T, Kesicki EA, Odingo J, Parish T. Validation of the essential ClpP protease in Mycobacterium tuberculosis as a novel drug target. J Bacteriol. 2012;194(3):663–8. doi: 10.1128/JB.06142-11 22123255 PMC3264079

[98] Saini V, Chinta KC, Reddy VP, Glasgow JN, Stein A, Lamprecht DA, et al. Hydrogen sulfide stimulates Mycobacterium tuberculosis respiration, growth and pathogenesis. Nat Commun. 2020;11(1):557. doi: 10.1038/s41467-019-14132-y 31992699 PMC6987094

[99] Rahman MA, Cumming BM, Addicott KW, Pacl HT, Russell SL, Nargan K, et al. Hydrogen sulfide dysregulates the immune response by suppressing central carbon metabolism to promote tuberculosis. Proc Natl Acad Sci U S A. 2020;117(12):6663–74. doi: 10.1073/pnas.1919211117 32139610 PMC7104411

[100] Sharrock A, Ruthe A, Andrews ESV, Arcus VA, Hicks JL. VapC proteins from Mycobacterium tuberculosis share ribonuclease sequence specificity but differ in regulation and toxicity. PLoS One. 2018;13(8):e0203412. doi: 10.1371/journal.pone.0203412 30169502 PMC6118392

[101] Sakowski ET, Koster S, Portal Celhay C, Park HS, Shrestha E, Hetzenecker SE, et al. Ubiquilin 1 Promotes IFN-γ-Induced Xenophagy of Mycobacterium tuberculosis. PLoS Pathog. 2015;11(7):e1005076. doi: 10.1371/journal.ppat.1005076 26225865 PMC4520715

[102] Dziadek B, Brzostek A, Grzybowski M, Fol M, Krupa A, Kryczka J, et al. Mycobacterium tuberculosis AtsG (Rv0296c), GlmU (Rv1018c) and SahH (Rv3248c) Proteins Function as the Human IL-8-Binding Effectors and Contribute to Pathogen Entry into Human Neutrophils. PLoS One. 2016;11(2):e0148030. doi: 10.1371/journal.pone.0148030 26829648 PMC4734655

[103] Boradia VM, Malhotra H, Thakkar JS, Tillu VA, Vuppala B, Patil P, et al. Mycobacterium tuberculosis acquires iron by cell-surface sequestration and internalization of human holo-transferrin. Nat Commun. 2014;5:4730. doi: 10.1038/ncomms5730 25163484

[104] Penn BH, Netter Z, Johnson JR, Von Dollen J, Jang GM, Johnson T, et al. An Mtb-Human Protein-Protein Interaction Map Identifies a Switch between Host Antiviral and Antibacterial Responses. Mol Cell. 2018;71(4):637-648.e5. doi: 10.1016/j.molcel.2018.07.010 30118682 PMC6329589

[105] Kruh-Garcia NA, Wolfe LM, Chaisson LH, Worodria WO, Nahid P, Schorey JS, et al. Detection of Mycobacterium tuberculosis peptides in the exosomes of patients with active and latent M. tuberculosis infection using MRM-MS. PLoS One. 2014;9(7):e103811. doi: 10.1371/journal.pone.0103811 25080351 PMC4117584

[106] Hood MI, Skaar EP. Nutritional immunity: transition metals at the pathogen-host interface. Nat Rev Microbiol. 2012;10(8):525–37. doi: 10.1038/nrmicro2836 22796883 PMC3875331

[107] Castillo E, Dekonenko A, Arko-Mensah J, Mandell M, Dupont N, Jiang S. Autophagy protects against active tuberculosis by suppressing bacterial burden and inflammation. Proc Natl Acad Sci U S A. 2012;109(46):E3168-76. 10.1073/pnas.1210500109 PMC3503152 23093667

[108] Gopalakrishnan A, Salgame P. Toll-like receptor 2 in host defense against Mycobacterium tuberculosis: to be or not to be-that is the question. Curr Opin Immunol. 2016;42:76–82. doi: 10.1016/j.coi.2016.06.003 27326654 PMC5086274

[109] Srivastava S, Grace PS, Ernst JD. Antigen Export Reduces Antigen Presentation and Limits T Cell Control of M. tuberculosis. Cell Host Microbe. 2016;19(1):44–54. doi: 10.1016/j.chom.2015.12.003 26764596 PMC4715867

[110] Grace PS, Ernst JD. Suboptimal Antigen Presentation Contributes to Virulence of Mycobacterium tuberculosis In Vivo. J Immunol. 2016;196(1):357–64. doi: 10.4049/jimmunol.1501494 26573837 PMC4684992

[111] Prados-Rosales R, Carreño LJ, Batista-Gonzalez A, Baena A, Venkataswamy MM, Xu J, et al. Mycobacterial membrane vesicles administered systemically in mice induce a protective immune response to surface compartments of Mycobacterium tuberculosis. mBio. 2014;5(5):e01921-14. doi: 10.1128/mBio.01921-14 25271291 PMC4196239

[112] Watson A, Li H, Ma B, Weiss R, Bendayan D, Abramovitz L, et al. Human antibodies targeting a Mycobacterium transporter protein mediate protection against tuberculosis. Nat Commun. 2021;12(1):602. doi: 10.1038/s41467-021-20930-0 33504803 PMC7840946

[113] Zheng W, LaCourse S, Song B, Singh D, Khanna M, Olivo J. Diagnosis of paediatric tuberculosis by optically detecting two virulence factors on extracellular vesicles in blood samples. Nat Biomed Eng. 2022;6(8):979–91. 35986185 10.1038/s41551-022-00922-1 PMC9391224

