# Supplementary figures and images for: Iron-restricted Mycobacterium tuberculosis exports pathogenicity factors packed in extracellular vesicles
Source: PLoS One. 2025 May 30;20(5):e0324919. doi: 10.1371/journal.pone.0324919 (PMC12124568; doi:10.1371/journal.pone.0324919)

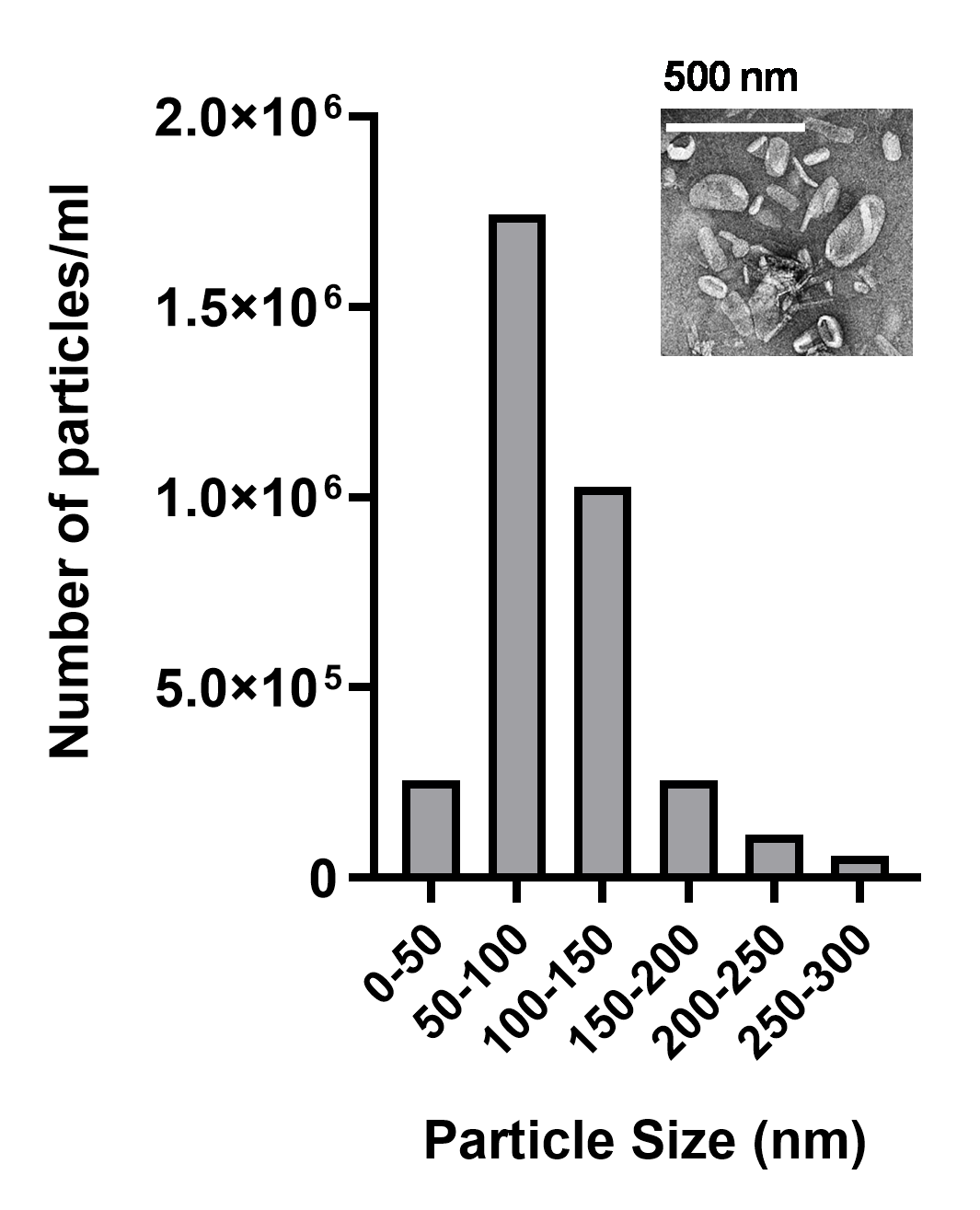

Supplement: S1 Fig — Purified MEVs were analyzed by electron microscopy, and size distribution was validated by nanoparticle tracking analysis (NTA) conducted using ZetaView (Particle Metrix). (TIF) [file pone.0324919.s004.tif]

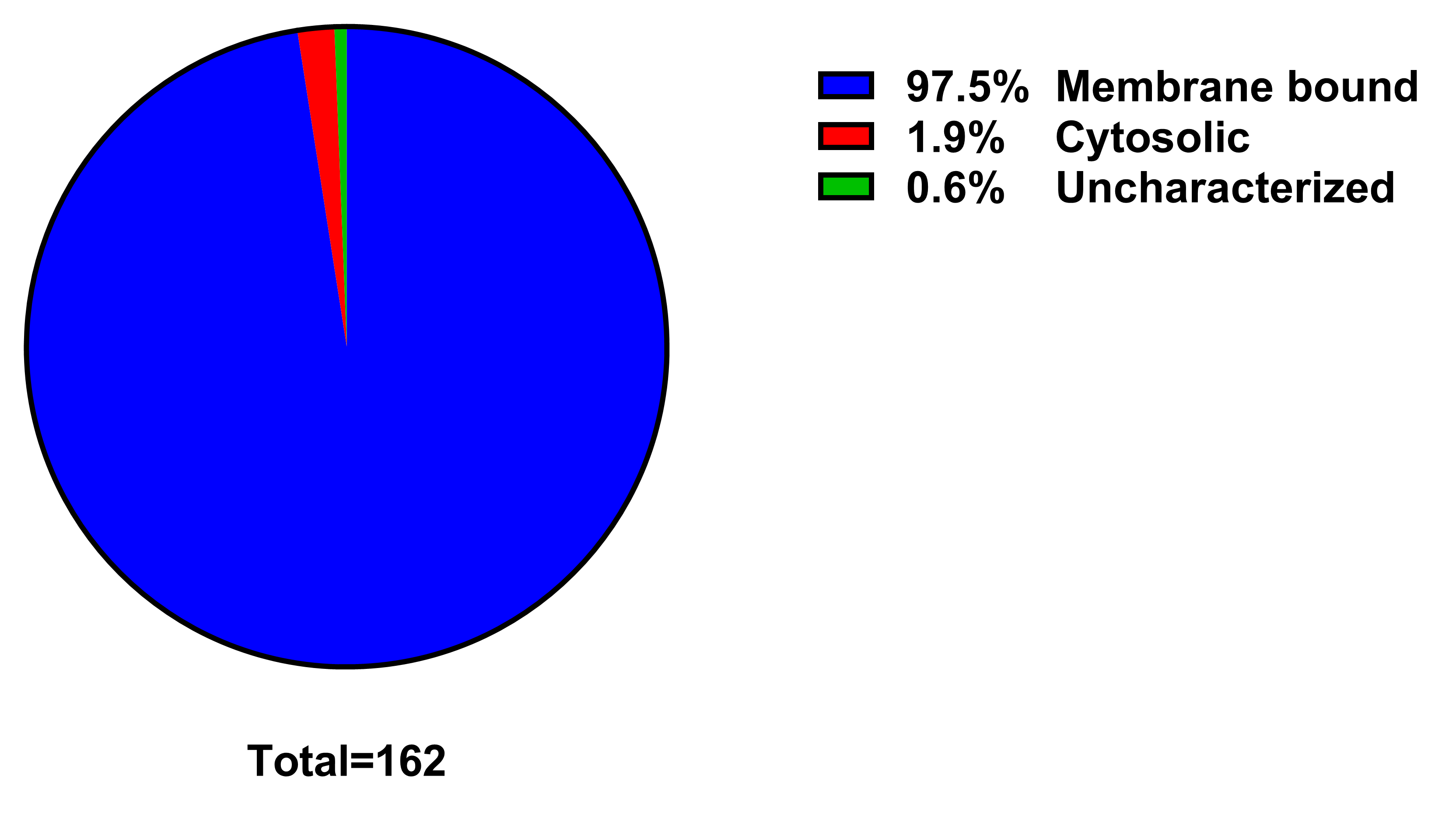

Supplement: S3 Fig — (TIFF) [file pone.0324919.s006.tiff]

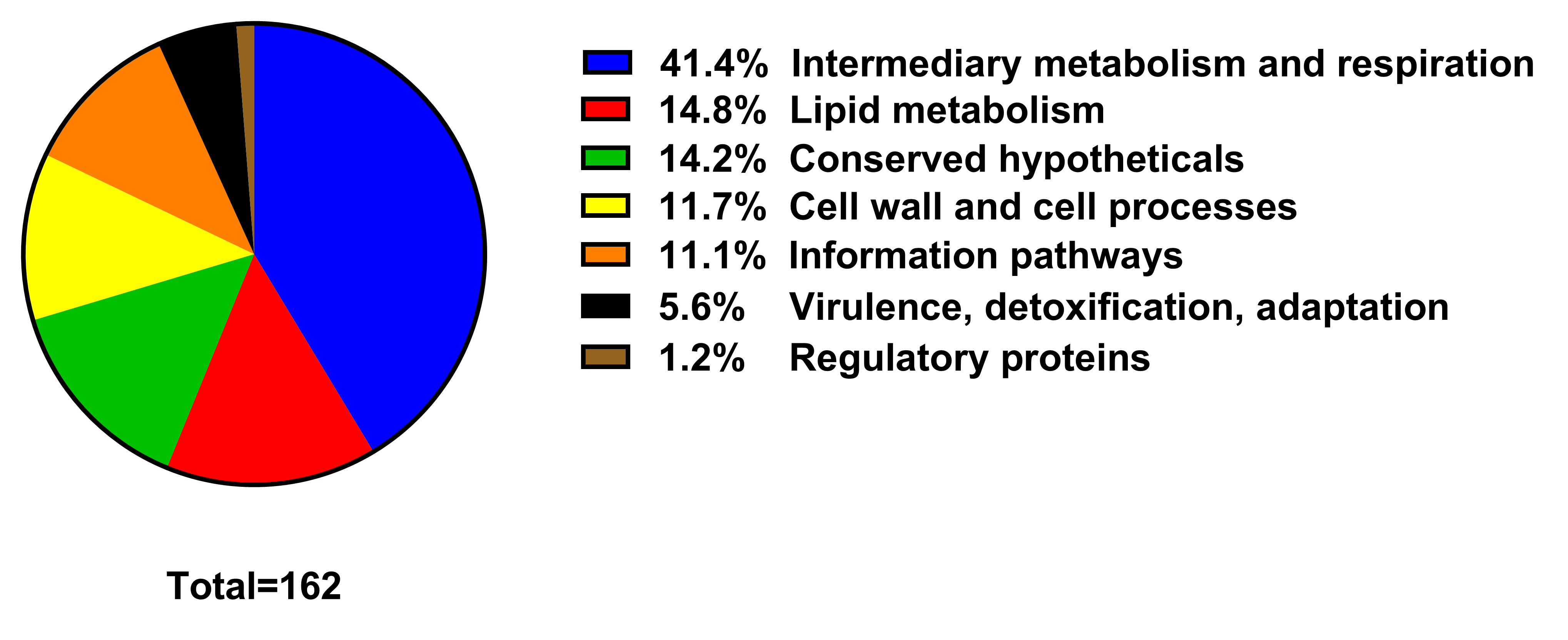

Supplement: S4 Fig — (TIFF) [file pone.0324919.s007.tiff]
