# Supplementary material for: Iron-restricted Mycobacterium tuberculosis exports pathogenicity factors packed in extracellular vesicles
Source: PLoS One. 2025 May 30;20(5):e0324919. doi: 10.1371/journal.pone.0324919 (PMC12124568; doi:10.1371/journal.pone.0324919)
Supplement: S2 Fig — (PDF) [file pone.0324919.s005.pdf]

Collect raw MS/MS intensity data for Cell pellet extracts and MEVs (n=3)

↓

Filter for proteins with 5 or more peptides in all three replicates

↓

Identify proteins using BLAST sequences (Uniprot)

↓

Annotate proteins using Mycobrowser, Uniprot, TB Data Base and cross reference with PubMed

↓

Designate cellular localization based on known location in proteomic studies of Mtb cell fractions and culture filtrates (Mycobrowser).
