# Supplementary material for: Iron-restricted Mycobacterium tuberculosis exports pathogenicity factors packed in extracellular vesicles
Source: PLoS One. 2025 May 30;20(5):e0324919. doi: 10.1371/journal.pone.0324919 (PMC12124568; doi:10.1371/journal.pone.0324919)
Supplement: S1 Table — (DOCX) [file pone.0324919.s008.docx]

**S1 Table. Proteins enriched in MEVs compared to whole cells.**

| **Protein** | **MW** | **IP** | **AA** | **Protein Family** | **Sequence Motifs** | **iBAQ MEVs/ CP** | **pValue** |
| --- | --- | --- | --- | --- | --- | --- | --- |
| GltA2 | 47 | 5.3 | 431 |  | FMN-binding domain | 1.355 | 0.004 |
| LpqH | 15 | 7.1 | 159 | LppX/LprAFG family | Lipoprotein lipid attachment site | 2.99 | <0.001 |
| LprG | 24 | 8.2 | 236 | LppX/LprAFG family | Lipoprotein lipid attachment site | 2.07 | <0.001 |
| FadD2 | 59 | 6.7 | 560 | ATP-dep-AMP binding enzyme |  | 1.77 | 0.001 |
| Rv0831c | 30 | 4.8 | 271 |  |  | 2.71 | <0.001 |
| Rv1288 | 49 | 5.4 | 456 |  |  | 4.52 | <0.001 |
| Rv1780 | 20 | 5.8 | 187 |  |  | 3.75 | 0.004 |
| PfkA | 36 | 6.6 | 343 |  |  | 2.24 | 0.001 |
| LpqN | 23 | 4.3 | 228 | LppX/LprAFG family | Lipoprotein lipid attachment site | 3.68 | <0.001 |
| Cds1 | 37 | 6.1 | 346 | Cystein synthase |  | 3.39 | 0.003 |
| Rv3205 | 31 | 6.8 | 292 |  |  | 3.22 | <0.001 |
| TopA | 102 | 8.7 | 934 |  |  | 3.2 | 0.001 |
| InhA | 28 | 6 | 269 | Enoyl-ACP reductases |  | 3.13 | <0.001 |
| RpsO | 10 | 11.5 | 89 |  |  | 3.11 | 0.004 |
| Rv0315 | 32 | 4.7 | 294 | GH16 domain-containing protein | Tat signal; Glycosyl hydrolases | 3 | 0.002 |
| RpsB | 31 | 6.3 | 287 |  |  | 2.93 | <0.001 |
| Rv1919c | 16 | 10.9 | 154 | Polyketide cyclase/dehydrase | PYR-PYL-RCRA | 2.89 | 0.001 |
| RpsN1 | 6 | 11.3 | 61 | ribosomal proteins S14p/S29e |  | 2.53 | <0.001 |
| Gdh | 176 | 5.5 | 1624 | NAD-glutamate dehydorgenas, bacteria | GDH domain | 2.53 | <0.001 |
| VapC10 | 14 | 11.4 | 133 | PINc/VapC | PIN domain | 2.52 | <0.001 |
| TesB2 | 31 | 6.4 | 281 | Acyl-CoA thioesterease C-terminal | HotDog fold | 2.41 | <0.001 |
| LpqE | 18 | 9.9 |  | LppX/LprAFG family | Lipoprotein lipid attachment site | 2.4 | 0.004 |
| LprF | 26 | 9.1 | 261 | LppX/LprAFG family | Lipoprotein lipid attachment site | 2.32 | <0.001 |
| Rv1769 | 45 | 7.9 | 414 | D-aminoacide metabolism enzyme | PLP dependent enzyme | 2.32 | <0.001 |
| Rv0247c | 28 | 6.1 | 248 | SDH/FRD-Iron sulfur | 2Fe-2S ferrodoxin | 2.26 | <0.001 |
| LprA | 24 | 5.2 | 244 | LppX/LprAFG family | Lipoprotein lipid attachment site | 2.22 | <0.001 |
| Rv2129c | 31 | 10.02 | 293 | Short chain dehydorgenase/reductase SDR | SDR domain | 2.07 | 0.005 |
| EchA21 | 29 | 4.7 | 274 | Enoyl-CoA hydratase | ECH domain | 2.06 | <0.001 |
| RplJ | 18 | 8.4 | 178 | Ribosomal proteins | SAP domain | 1.77 | 0.001 |
| RplE | 21 | 10.7 | 187 | Ribosomal proteins | EAL domain | 1.67 | 0.001 |
| CitA | 40 | 5.2 | 373 |  |  | 1.3 | 0.001 |

**MW:** Molecular Weight (KDa); **IP:** Isoelectric Point; **AA:** Number of Amino Acids.
